# Host defence peptides identified in human apolipoprotein B as promising antifungal agents

**DOI:** 10.1007/s00253-021-11114-3

**Published:** 2021-02-12

**Authors:** Eliana Dell’Olmo, Rosa Gaglione, Angela Cesaro, Valeria Cafaro, Wieke R. Teertstra, Hans de Cock, Eugenio Notomista, Henk P. Haagsman, Edwin J. A. Veldhuizen, Angela Arciello

**Affiliations:** 1 Department of Chemical Sciences, University of Naples Federico II, 80126 Naples, Italy; 2 Department of Biomolecular Health Sciences, Division of Infectious Diseases and Immunology, Section Molecular Host Defence, Faculty of Veterinary Medicine, Utrecht University, Utrecht, The Netherlands; 3 Istituto Nazionale di Biostrutture e Biosistemi (INBB), Rome, Italy; 4 Department of Biology, University of Naples Federico II, 80126 Naples, Italy; 5 Molecular Microbiology, Department of Biology, Faculty of Science, Utrecht University, Utrecht, The Netherlands

**Keywords:** Antifungal peptides, Human cryptides, Fungal infections, Peptide therapeutics

## Abstract

**Abstract:**

Therapeutic options to treat invasive fungal infections are still limited. This makes the development of novel antifungal agents highly desirable. Naturally occurring antifungal peptides represent valid candidates, since they are not harmful for human cells and are endowed with a wide range of activities and their mechanism of action is different from that of conventional antifungal drugs. Here, we characterized for the first time the antifungal properties of novel peptides identified in human apolipoprotein B. ApoB-derived peptides, here named r(P)ApoB_L_^Pro^, r(P)ApoB_L_^Ala^ and r(P)ApoB_S_^Pro^, were found to have significant fungicidal activity towards *Candida albicans* (*C. albicans*) cells. Peptides were also found to be able to slow down metabolic activity of *Aspergillus niger* (*A. niger*) spores. In addition, experiments were carried out to clarify the mechanism of fungicidal activity of ApoB-derived peptides. Peptides immediately interacted with *C. albicans* cell surfaces, as indicated by fluorescence live cell imaging analyses, and induced severe membrane damage, as indicated by propidium iodide uptake induced upon treatment of *C. albicans* cells with ApoB-derived peptides. ApoB-derived peptides were also tested on *A. niger* swollen spores, initial hyphae and branched mycelium. The effects of peptides were found to be more severe on swollen spores and initial hyphae compared to mycelium. Fluorescence live cell imaging analyses confirmed peptide internalization into swollen spores with a consequent accumulation into hyphae. Altogether, these findings open interesting perspectives to the application of ApoB-derived peptides as effective antifungal agents.

**Key points:**

- *Human cryptides identified in ApoB are effective antifungal agents*.
- *ApoB-derived cryptides exert fungicidal effects towards C. albicans cells*.
- *ApoB-derived cryptides affect different stages of growth of A. niger*.

Graphical abstract
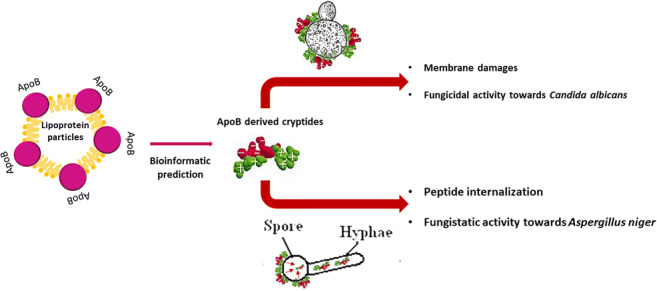

**Supplementary Information:**

The online version contains supplementary material available at 10.1007/s00253-021-11114-3.

## Introduction

The emergence of fungal infections represents a worldwide issue with a serious economic and social impact (Ciociola et al. 2016). Since the 1980s, an increase in cases of serious invasive fungal infections is observed due to the growing number of highly susceptible people, mainly immunocompromised, elderly and transplanted subjects, cancer patients and premature infants (Brown et al. 2012). Fungal pathogens as *Candida*, *Aspergillus*, *Pneumocystis*, and *Cryptococcus* spp. are responsible for 1.4 million deaths each year (Brown et al. 2012; Sanglard 2016). The situation is even more complicated because of the variety and non-specificity of symptoms (Schmiedel and Zimmerli 2016), ranging from either mild and superficial (e.g*.* cutaneous infections as dermatophytosis and tinea versicolor) to life-threatening, systemic illness (e.g*.* candidiasis, aspergillosis and mucomycosis).

### Infections by *Candida* and *Aspergillus* species

Although *C. albicans* is a normal commensal for humans (Hancock and Lehrer 1998; Bennett et al. 2014), it is responsible for 75% of women vaginal candidiasis and for 90% of oropharyngeal candidiasis in HIV-infected patients with AIDS (Staab 1999; Sobel 2007). Indeed, *C. albicans* has been found to be able to invade a local site (mucocutaneous or cutaneous candidiasis, onychomycosis) or to cause systemic infections (renal, liver abscess, lung and nervous central system) (Badiee and Hashemizadeh 2014). It has also been reported that *Candida* species are the most common pathogens responsible for infections in hospitalized patients characterized by a high mortality rate (Strollo et al. 2017). Among fungal species able to colonize humans, *Aspergillus* spp*.* have been reported to be responsible for several types of infections and allergic bronchopulmonary aspergillosis (Anaissie et al. 2002; Alastruey-Izquierdo et al. 2013; Badiee and Hashemizadeh 2014). Indeed, *Aspergillus* ubiquitous spores are able to reach the respiratory tract by inhalation, thus causing noninvasive and invasive pulmonary aspergillosis, the latter especially in immunocompromised hosts. Along with the difficulties in the diagnosis, the narrow spectrum of available antifungals leads to the rapid rise of resistance phenotypes (Cowen et al. 2015; Fisher et al. 2018a). Indeed, the therapeutic options for invasive fungal infections are limited to only three structural classes of drugs, such as polyenes, azoles and echinocandins (Morita and Nozawa 1985; Roemer and Krysan 2014). Polyenes, including amphotericin B, exert significant toxic effects that might be overcome by developing awfully expensive formulations. For these reasons, the most tolerated azoles or echinocandins are the preferred therapeutic option (Bellmann and Smuszkiewicz 2017). Examples are represented by fluconazole to treat *Candida* infections or voriconazole to treat aspergillosis. Echinocandins are effective against several fungal strains, such as *Candida* spp., including no-*albicans* strains, and have been reported to exert fungistatic effects on *Aspergillus* spp. (Bowman et al. 2002; Bellmann and Smuszkiewicz 2017). Unfortunately, many cases of resistant fungal strains have been reported, due to the lower number of antifungal drugs available and to delays in diagnosis (Cowen et al. 2015; Fisher et al. 2018b). Hence, the discovery and development of novel alternative strategies is imperative.

### Host defence peptides as antifungal peptides

Host defence peptides (HDPs), which comprise antifungal peptides (AFPs), may represent valid candidates (Sun et al. 2018; Mookherjee et al. 2020) (Zasloff 2002; Thevissen et al. 2007; Lupetti et al. 2008) because of their unique properties, such as their selectivity towards bacterial/fungal cells, their mechanism of action, which is different from that of conventional antifungal drugs and their moderate toxicity and immunogenicity (Lupetti et al. 2008; Aoki and Ueda 2013; Ciociola et al. 2016). Antifungal peptides represent a group of evolutionarily conserved molecules of the innate immune system present in all complex living organisms. They are characterized by common features, such as small size, positive net charge and high hydrophobicity (Thery et al. 2019a). Based on their mechanism of action, they can be classified into (i) membrane traversing peptides, which are able to lead membrane pore formation or to act on specific targets, such as β-glucan or chitin synthesis; and (ii) non-membrane traversing peptides, which interact with cell membrane and consequently cause cell lysis (Neelabh et al. 2016). Nowadays, more than 1200 antifungal peptides isolated from bacteria, other fungi, plants and animals have been identified and listed in databases (Essig et al. 2014; Thery et al. 2019a). Mammalian organisms release a large amount of AFPs as components of the innate immune system. Examples are α- and β-defensins, cathelicidins and histatins (Kościuczuk et al. 2012; Cuperus et al. 2013; Bondaryk et al. 2017), which have been found to be effective towards a wide range of fungal pathogens (Mookherjee et al. 2020), including *C. albicans* and *Aspergillus spp.*

### ApoB-derived peptides as antifungal peptides

Here, we analysed for the first time the antifungal properties of three recombinant peptides identified in human apolipoprotein B (Gaglione et al. 2017; Gaglione et al. 2019b; Gaglione et al. 2019a), here named r(P)ApoB_L_^Pro^, r(P)ApoB_L_^Ala^ and r(P)ApoB_S_^Pro^, where (P) indicates the presence of an additional Pro residue at the N-terminus of the peptides released by the acidic cleavage of an Asp-Pro bond; superscripts “Pro” and “Ala” stand for the amino acid residue at position 7 (Gaglione et al. 2020), whereas subscripts “L” and “S” indicate a longer and a shorter version of the identified peptide, respectively (Gaglione et al. 2017; Gaglione et al. 2020). Peptides’ sequences, lengths, isoelectric points, experimental and theoretical molecular weights and net charges at neutral pH are reported in Table 1. In the present study, we found that ApoB-derived peptides exert significant fungicidal effects towards *C. albicans* cells by affecting membrane permeability. ApoB-derived peptides have been found to inhibit *A. niger* filamentous fungus strongly also. Indeed, peptides were demonstrated to slow down the metabolic activity of *A. niger* spores, hyphae and branched mycelium by internalization into germinating spores and consequent accumulation into hyphae. Our results strongly suggest that ApoB-derived peptides represent valid candidates for the development of novel antifungal agents.

**Table 1:** Peptides’ sequences, lengths, isoelectric points, experimental and theoretical molecular weights, and net charges at neutral pH

Peptide	Sequence	Length	Experimental M*r*	Theoretical M*r*	Isoelectric point	Net charge at neutral pH
r(P)ApoB_L_^Pro^	**P**HVALKPGKLKFIIPSPKRPVKLLSGGNTLHLVSTTKT	37	4076.96 Da	4074.96 Da	11.4	7.2
r(P)ApoB_L_^Ala^	**P**HVALKAGKLKFIIPSPKRPVKLLSGGNTLHLVSTTKT	37	4044.75 Da	4048.92 Da	11.4	7.2
r(P)ApoB_S_^Pro^	**P**HVALKPGKLKFIIPSPKRPVKLLSG	26	2820.85 Da	2821.54 Da	11.3	6.1
r(C)ApoB_L_^Pro^	**C**HVALKPGKLKFIIPSPKRPVKLLSGGNTLHLVSTTKT	37	4465.64 Da	4468.28 Da	10.69	7.1

## Materials and methods

### Materials

All reagents were purchased from Merck (Milan, Italy), unless specified otherwise. CATH-2 peptide was obtained from CPC Scientific Inc. (Sunnyvale, USA).

### Fungal strains and growth conditions

Cultures of *C. albicans* ATCC 10231 were grown on Yeast Malt (YM) agar plates. For all the experiments, yeasts were cultured at 30 °C in 10 mL yeast extract peptone dextrose broth (YPD) until mid-logarithmic phase was reached. Growth rate was monitored by measuring optical density (OD) values at 620 nm; when mid-logarithmic phase was reached, *Candida* cells were collected and diluted to 2 × 10^6^ CFU/mL in 1/100 YM broth. To determine minimal fungicidal concentration (MFC) values, tenfold dilutions of culture were plated into YM broth. *A. niger* N402 was grown at 30 °C in 20-mL minimal medium (MM) (De Vries et al. 2004) containing 2% glucose and 1.5% agar. Conidia used to inoculate cultures were harvested from 4-day-old colonies by using a solution containing 0.8% NaCl and 0.005% Tween-80.

### Expression ad isolation of recombinant ApoB-derived peptides

Expression and isolation of recombinant peptides were performed as described previously (Gaglione et al. 2017; Pane et al. 2018b; Gaglione et al. 2019b; Gaglione et al. 2020). ApoB-derived peptides’ sequences are reported in Table 1.

### Production of fluorescently labelled r(C)ApoB_L_^Pro^

r(C)ApoB_L_^Pro^ was obtained by chemical hydrolysis of purified ONC-DCless-H6-(C)-ApoB_L_^Pro^ fusion protein in 5 M guanidine-HCl containing 1 mM TCEP (tris(2-carboxyethyl)phosphine) at pH 7.4. A chimeric construct was expressed and purified as previously reported (Gaglione et al. 2017; Pane et al. 2018b; Gaglione et al. 2019b; Gaglione et al. 2020). Peptide release was monitored by reversed-phase high-performance liquid chromatography (RP-HPLC) carried out by using a Jasco LC-4000 system equipped with PU-4086 semipreparative pumps and MD-4010 photo diode array detector. A Europa Protein 300 C18 column (5 μm, 25 × 1) from Teknokroma (Barcelona, Spain) was used. Solvents were 0.05% trifluoroacetic acid (TFA) in water (solvent A) and 0.05% TFA in acetonitrile (solvent B). Elution profiles were recorded by a linear gradient as follows: from 5 to 25% solvent B in 10 min, from 25 to 35% solvent B in 30 min, from 35 to 50% solvent B in 10 min, from 50 to 100% solvent B in 10 min, and isocratic elution at 100% solvent B for 10 min. Elution was monitored at 214 nm at a flow rate of 2 mL/min. r(C)ApoB_L_^Pro^ peptide was then purified by a column-free procedure based on different solubilities of carrier and peptide at pH 7.0 (Pane et al. 2018b). To this purpose, the hydrolysis mixture was neutralized by adding a diluted ammonia solution for 5 min at 28 °C under nitrogen atmosphere. Insoluble fusion protein and carrier were then separated from soluble peptide by 10-min centrifugation at 18,000 g at 4 °C. The soluble fraction was analysed by RP-HPLC performed by using a Europa Protein 300 C18 column (Teknokroma, Barcelona, Spain) as previously described, in order to evaluate peptide purity. Supernatant, containing soluble peptide, was subjected to the labelling reaction.

### Labelling of purified peptide

r(C)ApoB_L_^Pro^ N-terminal cysteine reactive residue was labelled with the thiol-reactive probe 5-iodoacetamidofluorescein (5’-IAF), in order to produce 5’-IAF-r(C)ApoB_L_^Pro^ labelled peptide. Purified r(C)ApoB_L_^Pro^ peptide (9.6 mg, 60 μM final concentration) was incubated with 5’-IAF (0.25 mM final concentration; 15 mM stock solution in dimethyl formamide) in 15 mM sodium phosphate buffer (NaP) pH 7.4 containing 2 M guanidine-HCl for 2 h at 25 °C in the dark under nitrogen atmosphere. Molar ratio of 5’-IAF over thiols was 4:1. r(C)ApoB_L_^Pro^ labelling reaction was monitored by RP-HPLC performed on a Europa Protein 300 C18 column as reported above (Figure S2). To simplify peptide purification by RP-HPLC, the reaction was quenched by adding L-cysteine in a molar excess of 10:1 on 5’-IAF for 1 h at 37 °C in the dark. 5’IAF-r(C)ApoB_L_^Pro^ was purified by RP-HPLC and lyophilized and resuspended in water. Labelled peptide concentration was determined using the molar extinction coefficient reported in the literature (5’-IAF ε492 nm = 80,000–85,000 M^−1^ cm^−1^) and by BCA colorimetric assay. Purity of labelled peptide was evaluated by RP-HPLC performed on Europa Protein 300 C18 column.

### Determination of minimal fungicidal concentration values

MFC (minimum fungicidal concentration) values were assessed by colony counting assays, as previously described (Van Dijk et al. 2007), with few modifications. Briefly, 50 μL of a 2 × 10^6^ CFU/mL suspension of *C. albicans* ATCC 10231 cells in 1/100 YM broth were incubated for 3 h at 37 °C with an equal volume of peptide (0–40 μM). Tenfold dilutions in YM broth were then plated onto YPD agar plates and incubated overnight at 37 °C. Finally, colonies of surviving yeast cells were counted.

### Killing kinetic studies

To kinetically analyse fungicidal activity of ApoB-derived peptides, experiments on *C. albicans* ATCC 10231 cells were performed. Yeast cells grown overnight in YM (Yeast Malt) medium were diluted in fresh YM medium and then incubated at 37 °C until logarithmic phase of growth was reached. Yeasts were then diluted to 2 × 10^6^ CFU/mL in a final volume of 500 μL in 1/100 YM broth and mixed with the peptides (1:1 v/v). Increasing concentrations of peptide were analysed (ranging from 0 to 20 μM). At defined time intervals, samples (20 μL) were serially diluted (from 10- to 10,000-fold), and 100 μL of each dilution was plated on YPD Agar. Following an incubation of 16 h at 37 °C, yeast colonies were counted.

### PI uptake assay

Propidium iodide uptake was monitored as previously described (Stone et al. 2003) with some modifications. Briefly, 45 μL of 1 × 10^7^ CFU/mL of *C. albicans* ATCC 10231 cells were plated into 96-well plates, treated with 45 μL of peptides with increasing concentrations (0–20 μM) and incubated for 1 h at 37 °C. After that, 10 μL of PI at a final concentration of 5 μM was added. After 10 min of incubation, PI fluorescence was measured by using a microtiter plate reader (FLUOstar Omega, BMG LABTECH, Germany) at an excitation wavelength of 485 nm and an emission wavelength of 650 nm. The percentage of PI uptake was calculated as follows: [F(sample)-F(CTRL)/F(100%) − F(CTRL)] × 100, where F(CTRL) is the fluorescence of untreated sample and F(100%) is the fluorescence of heat-treated samples (15 min at 95 °C).

### ATP release assay

ATP released by cells exposed to peptides was measured by using an ATP determination kit from Molecular Probes (Life Sciences, Bleiswijk, The Netherlands). Briefly, a suspension of 1 × 10^7^ CFU/mL *C. albicans* ATCC 10231 cells in 1:100 YM was incubated with increasing concentrations of each peptide for 10 and 60 min at 37 °C. Samples were then centrifuged for 1 min at 1200×*g* and the supernatant was stored on ice for ATP determination, performed as described by the manufacturer. ATP concentration in control samples was found to be lower than 10 nM (data not shown).

### Localization of 5’-IAF-r(C)ApoB_L_^Pro^ by confocal laser scanning live imaging microscopy

Experiments were performed as previously described by Jang and co-workers with some modifications (Jang et al. 2010). For all the experiments, 35-mm culture dishes (FluoroDish™, WPI, Sarasota, FL) were coated with 0.5 mg/mL Concanavalin A in water. A suspension (100 μL) of 1 × 10^7^ CFU/mL *C. albicans* ATCC 10231 in 1:100 YM medium was then added. Fluorescently labelled peptide (50 μL) was added in the medium containing 5 μM PI. In the case of *A. niger* N402 hyphae, 100 μL of a solution of 1 × 10^7^ spores/mL were incubated at 37 °C for 24 h in the presence of peptides. At defined time points (0, 16 and 24 h), analyses were performed. Images were acquired by using a Leica SPE-II and a 63× objective at the Centre for Cell Imaging (CCI)—Utrecht University. A 488-nm argon laser and a 561-nm DPSS laser were used for simultaneous detection of 5’-IAF-r(C)ApoB_L_^Pro^ and PI, respectively.

### Metabolic activity analyses

The effects of ApoB-derived peptides on *A. niger* N402 metabolic activity were analysed by using cell proliferator reagent WST-1 (Roche Applied Science, Mannheim, Germany). Briefly, 45 μL of a 1 × 10^5^ spores/mL suspension in MM were incubated for 24 h at 30 °C with an equal volume of peptide (0–40 μM). In each well, 10 μL of WST-1 10× were added. At defined time intervals, sample absorbance values were measured at 450 nm by using 650 nm as reference wavelength at a microtiter plate reader (FLUOstar Omega, BMG LABTECH, Germany). To investigate the effects of ApoB-derived peptides on *A. niger* N402 swollen spores, hyphae, and mycelium, these were incubated in MM containing 2% glucose for 6, 16 and 24 h prior to treatment with peptides, respectively.

### Statistical analyses

Statistical analysis was performed using ANOVA or Student’s *t* test. Significant differences were indicated as *(*P* < 0.05), **(*P* < 0.01), ***(*P* < 0.001) or ****(*P* < 0.0001).

## Results

### Antifungal activity of r(P)ApoB_L_^Pro^, r(P)ApoB_S_^Pro^ and r(P)ApoB_L_^Ala^ peptides

**Fig. 1:**
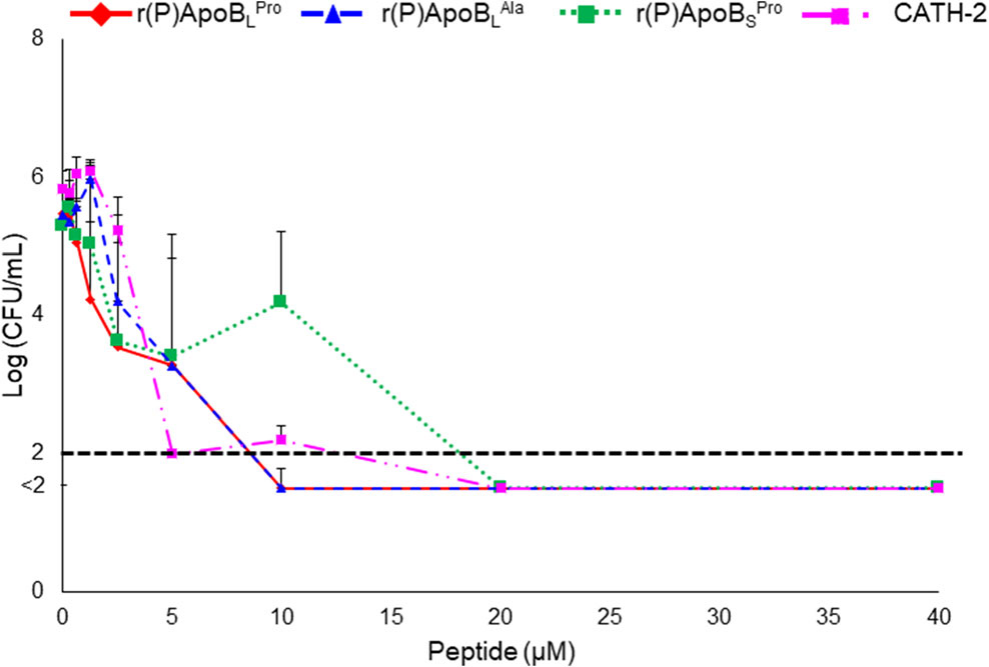
Antifungal activity of r(P)ApoB_L_^Pro^, r(P)ApoB_L_^Ala^ and r(P)ApoB_S_^Pro^ peptides. Minimum fungicidal concentration (MFC) values were assessed by colony count assays. Data represent the mean (± SEM) of three independent experiments, each one carried out with triplicate determinations. CATH-2 peptide was used as a positive control. Point values below the black dashed line represent conditions in which 0 colonies were counted

The antifungal properties of recombinant ApoB-derived peptides were tested. First of all, we evaluated purity and integrity of the peptides by performing SDS-PAGE and mass spectrometry analyses, as shown in Supplementary Figures S1 and S2. We firstly investigated the effects of peptides on *C. albicans* ATCC 10231. For this purpose, increasing amounts of r(P)ApoB_L_^Pro^, r(P)ApoB_S_^Pro^ or r(P)ApoB_L_^Ala^ were incubated with the fungal cells for 3 h and the chicken CATH-2 peptide was used as positive control. As shown in Fig. 1, ApoB-derived peptides were found to exert a strong fungicidal activity towards *C. albicans* ATCC 10231 at 10 μM of r(P)ApoB_L_^Pro^ and r(P)ApoB_L_^Ala^ and at 20 μM for the shorter version of the peptide. MFC_100_ (minimal fungicidal concentrations) values determined when peptides were tested on *C. albicans* ATCC 10231 are reported in Supplementary Table S1.

**Fig. 2:**
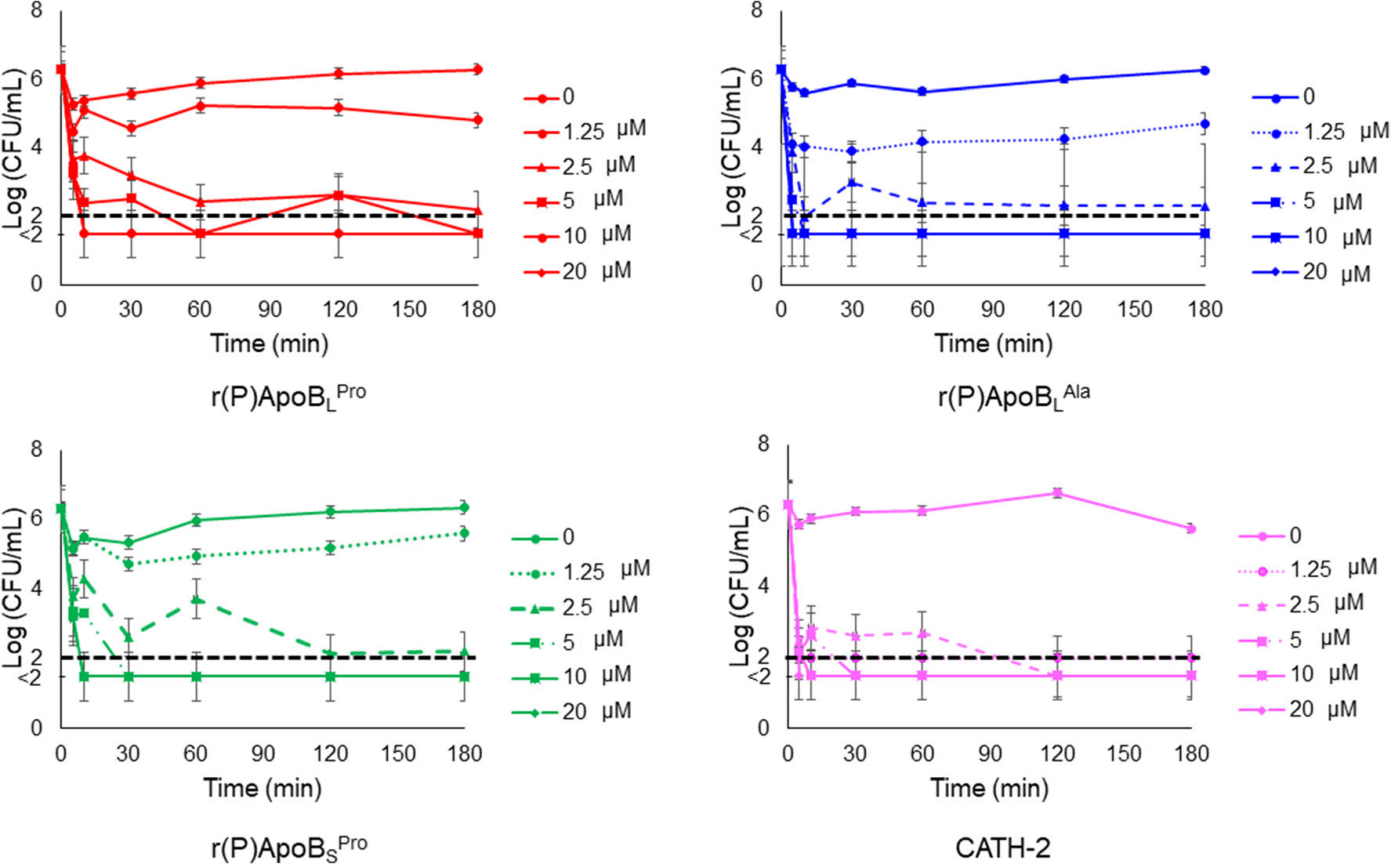
Time killing curves obtained by incubating *C. albicans* ATCC 10231 with increasing concentrations of r(P)ApoB_L_^Pro^, r(P)ApoB_L_^Ala^ or r(P)ApoB_S_^Pro^ peptides for different time intervals. Data represent the mean (± SEM) of at least three independent experiments, each one carried out with triplicate determinations. Point values below the black dashed lines represent conditions in which 0 colonies were counted

In *order* to analyse the antifungal effects of ApoB-derived HDPs over time, kinetic killing curves were obtained by treating *C. albicans* ATCC 10231 with increasing concentrations of each peptide and for different time intervals. At the highest peptide concentrations tested (5-10 μM), *C. albicans* ATCC 10231 was killed within 10 min, whereas, at lower concentrations (5 μM), the same effects were observed after 180 min (Fig. 2).

### Effects of ApoB-derived peptides on yeast cell membranes

**Fig. 3:**
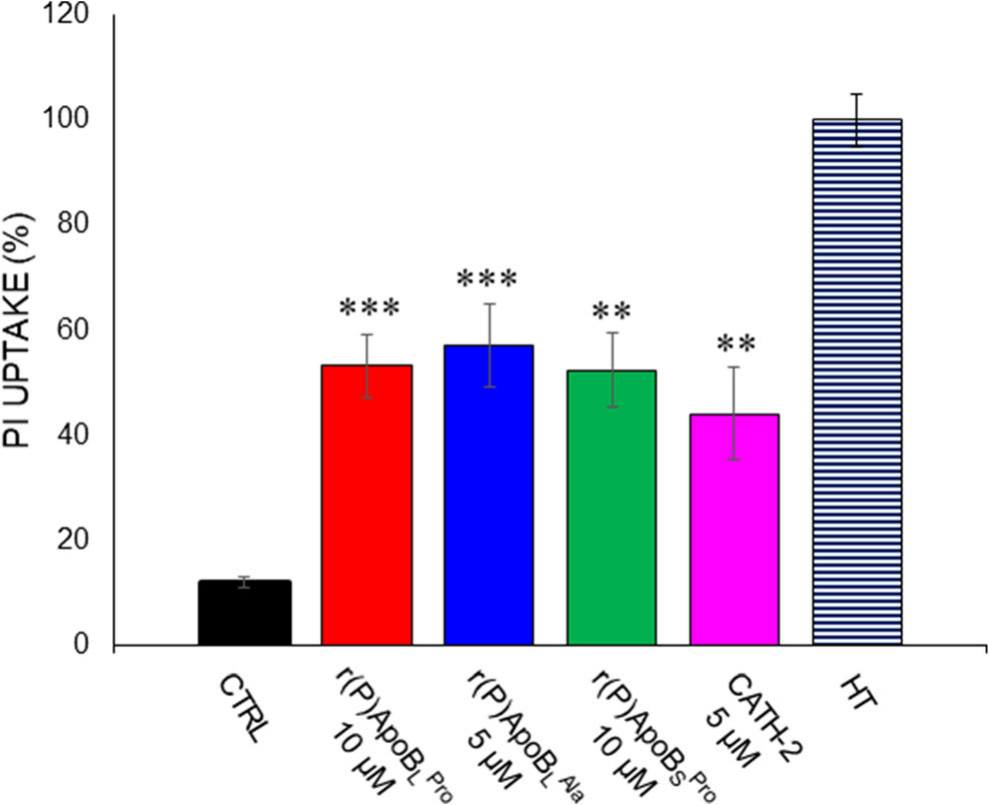
Propidium iodide (PI) uptake into *C. albicans* ATCC 10231 cells upon treatment with r(P)ApoB_L_^Pro^, r(P)ApoB_L_^Ala^ or r(P)ApoB_S_^Pro^. PI uptake was determined by a spectrofluorometric assay. Data represent the mean (± SEM) of at least three independent experiments, each one carried out with triplicate determinations. Significant differences were found to be ****P < 0.0001 for treated *versus* control samples. CATH-2 peptide and the heat-treated cells were used as positive controls

**Fig. 4:**
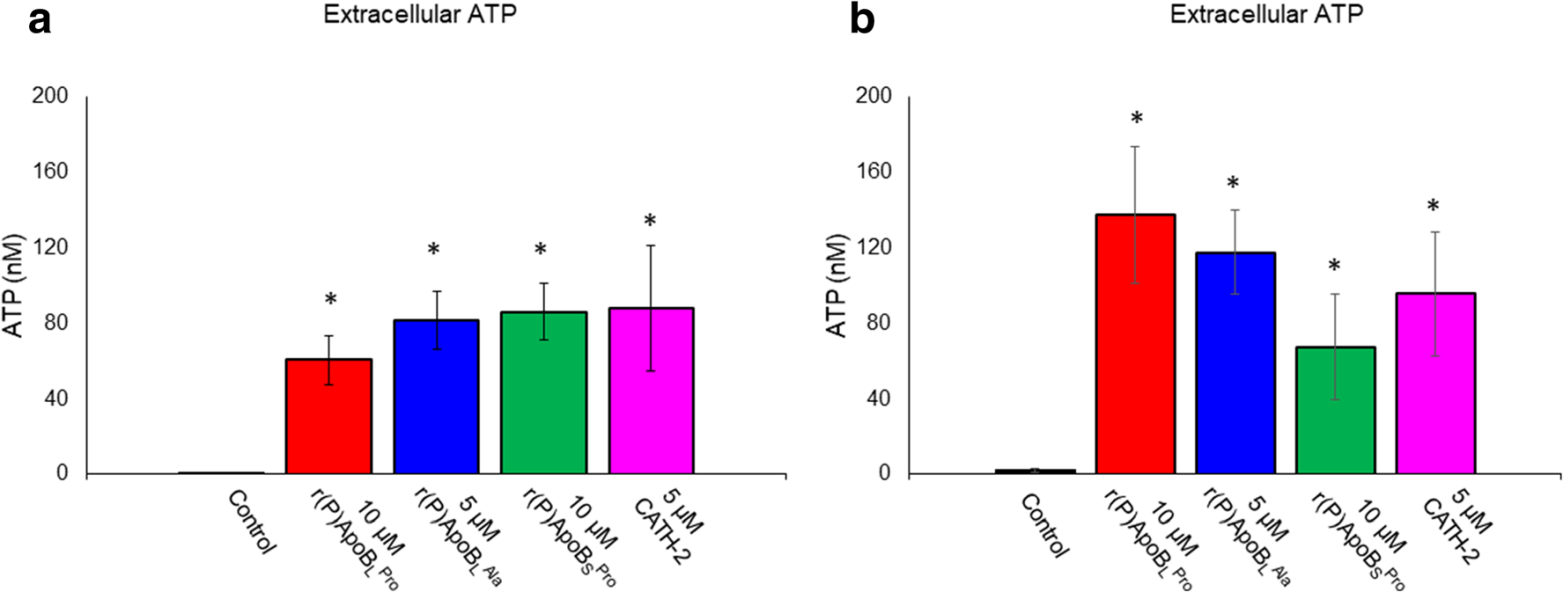
ATP release from *C. albicans* ATCC 10231 cells upon treatment with r(P)ApoB_L_^Pro^, r(P)ApoB_L_^Ala^ or r(P)ApoB_S_^Pro^. ATP release was determined in the culture medium. Data represent the mean (± SEM) of at least three independent experiments, each one carried out in duplicate, upon an incubation of 10 (A) or 60 min (B) with each peptide. Significant differences were found to be *P < 0.05 for treated *versus* control samples. CATH-2 peptide was used as a positive control

In order to determine the mechanism of fungicidal activity of ApoB-derived HDPs, propidium iodide uptake was analysed upon treatment of *C. albicans* ATCC 10231 cells with peptides. *C. albicans* ATCC 10231 cells were treated with ApoB-derived peptides at concentrations corresponding to MFC values for 1 h at 37 °C. A significant increase of propidium iodide uptake was selectively observed in the case of cells treated with ApoB-derived peptides (Fig. 3). This clearly suggests that ApoB-derived peptides’ antifungal activity against *C. albicans* ATCC 10231 cells was mediated by membrane permeabilization. To support this finding, the effects of ApoB-derived peptides on ATP leakage were also evaluated. To this purpose, *C. albicans* ATCC 10231 cells were treated with ApoB-derived peptides at concentrations corresponding to MFC values for 10 and 60 min at 37 °C. Interestingly, it was observed that r(P)ApoB_L_^Pro^, r(P)ApoB_L_^Ala^ and r(P)ApoB_S_^Pro^ peptides induce ATP release from *C. albicans* ATCC 10231 cells after 10 min of incubation, with a slight increase after 60-min incubation only in the case of r(P)ApoB_L_^Pro^ (Fig. 4). These results support propidium iodide data reported in Fig. 4 and indicate that membrane permeabilization occurs upon peptide treatment. Altogether, these findings allow us to hypothesize a fast interaction between ApoB-derived peptides and *C. albicans* ATCC 10231 cell membranes.

### Intracellular localization of ApoB-derived peptides in *C. albicans* ATCC 10231 cells

In order to further investigate the mechanism of action of ApoB-derived peptides, live imaging analyses were carried out. To this purpose, *C. albicans* ATCC 10231 cells were treated with a fluorescent version of r(P)ApoB_L_^Pro^ peptide, here named 5’-IAF-r(C)ApoB_L_^Pro^ (see the “Materials and methods” section for details) (Pane et al. 2018b; Pane et al. 2018a). The effects of 5’-IAF-r(C)ApoB_L_^Pro^ on *C. albicans* ATCC 10231 cells were found to be identical to those of r(C)ApoB_L_^Pro^, as reported in Supplementary Table S1 and in Supplementary Figure S3, thus indicating that the labelling procedure does not alter the peptide’s mechanism of action.

**Fig. 5:**
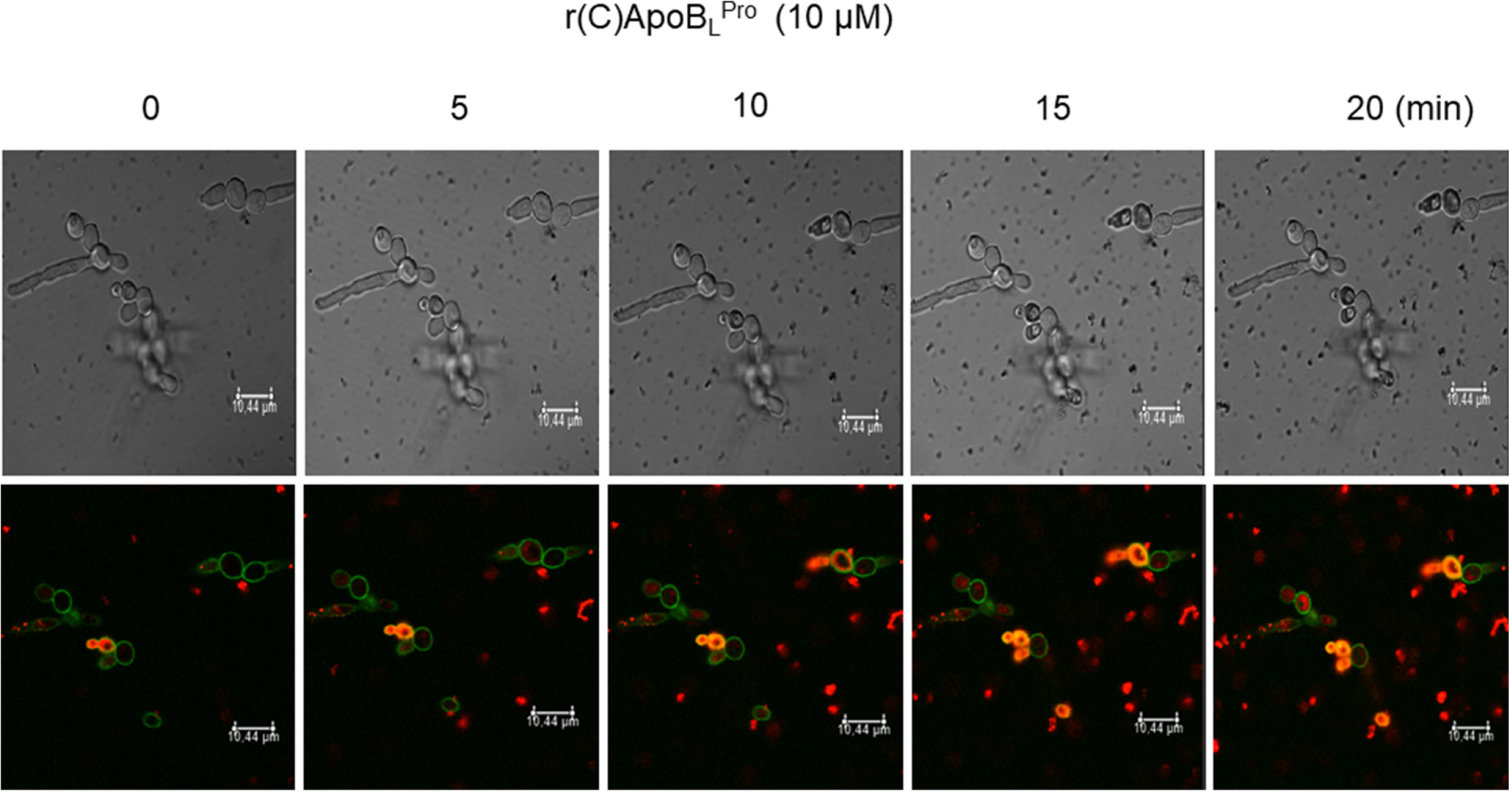
Localization of 5’-IAF-r(C)ApoB_L_^Pro^ peptide (green signal) into *C. albicans* ATCC 10231 cells in the presence of propidium iodide (PI) dye (red signal) analysed by confocal laser scanning live imaging microscopy. Movies are provided as Supplementary material. Scale bar10 μm

*C. albicans* ATCC 10231 cells were treated with 10 μM 5’-IAF-r(C)ApoB_L_^Pro^ for 30 min in the presence of propidium iodide dye. Upon treatment, *C. albicans* ATCC 10231 cells were analysed by confocal laser scanning live imaging microscopy. Interestingly, fluorescently labelled peptide (green signal in Fig. 5) immediately appeared and localized at the fungal surface, with a progressive increase of fluorescent signal intensity over time (Fig. 5). Real tracking of 5’-IAF-r(C)ApoB_L_^Pro^ is provided as a supplementary movie. In agreement with this, a progressive uptake of propidium iodide in treated cells was observed over time (red signals associated to *Candida* cells). The phenomenon appears clearly evident even if red spots, probably due to aggregation of PI in culture medium, appear visible outside the cells. This suggests that peptide interaction with yeast membranes is immediately responsible for severe membrane damages (Fig. 5).

### Analysis of ApoB-derived peptides effects on *A. niger* N402

**Fig. 6:**
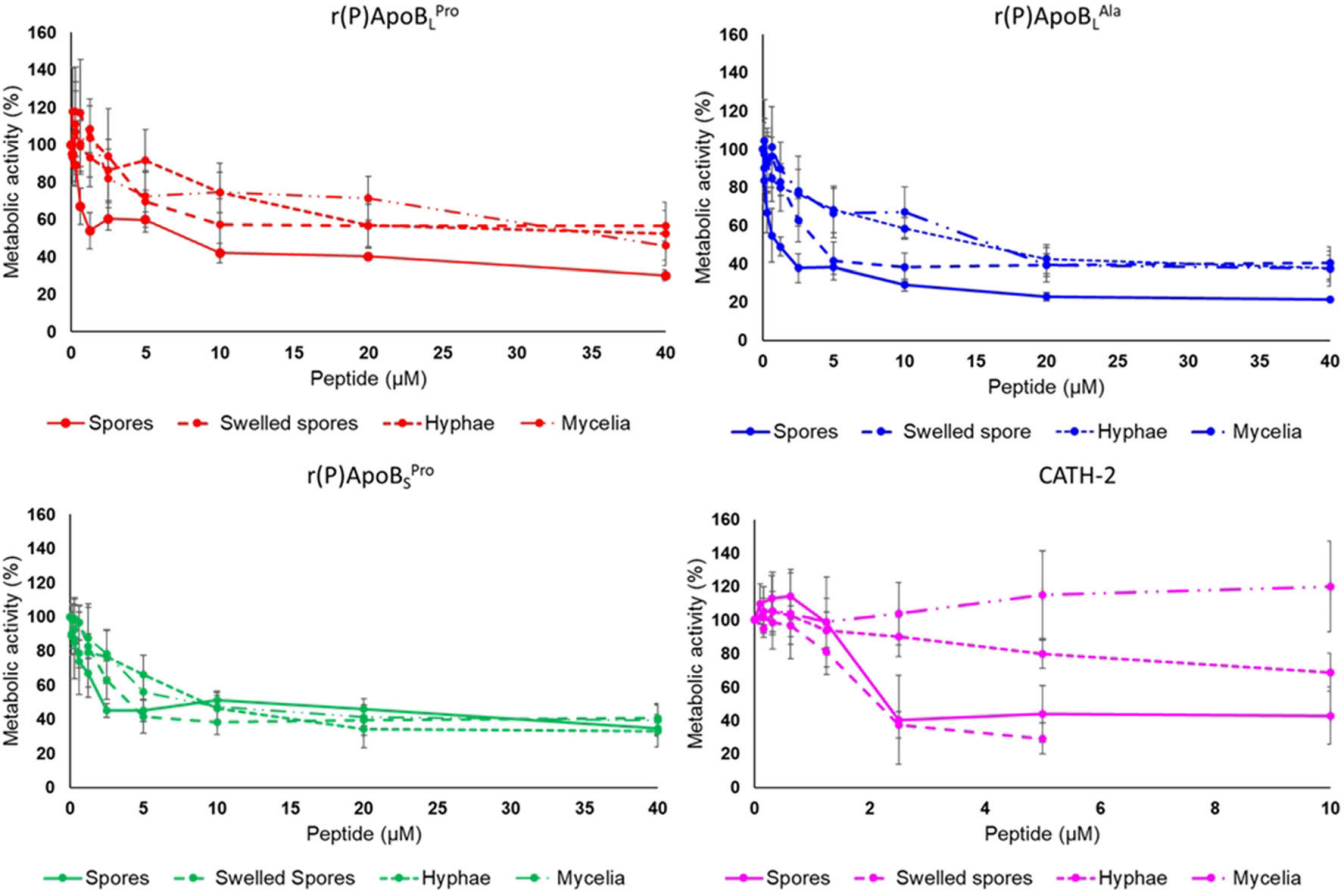
Dose response curves reporting the effects of r(P)ApoB_L_^Pro^, r(P)ApoB_L_^Ala^, r(P)ApoB_S_^Pro^ and CATH-2 used as a positive control, on the metabolic activity of *A. niger* N402 spores, swollen spores, initial hyphae and mycelium. Data represent the mean (± SEM) of at least three independent experiments, each one carried out in triplicate. Statistical analyses revealed significant differences between treated and control samples (*P < 0.05, ** for *P* < 0.01, ***P < 0.001 and ****P < 0.0001)

The effects of ApoB-derived peptides were also tested on the filamentous fungus *A. niger* N402. To this purpose, metabolic activity of fungal spores in the absence or in the presence of increasing concentrations of peptides was evaluated by performing WST-1 assays. Interestingly, r(P)ApoB_L_^Pro^, r(P)ApoB_L_^Ala^, r(P)ApoB_S_^Pro^ and CATH-2 peptides were found to reduce *A. niger* N402 spores’ metabolic activity in a dose-dependent manner (Fig. 6). On the basis of the results of metabolic activity assays, MFC_50_ (minimal fungicidal concentrations) values were calculated for *A. niger* spores as the peptide concentration required to inhibit the metabolic activity of spores by 50%. Data are reported in Supplementary Table S1. Filamentous fungi like *A. niger* reproduce asexually and form spores (or conidia) which easily spread in the environment. Spores are metabolically dormant and germinate under favourable environmental conditions (van Leeuwen et al. 2013). The switch from dormant spores to mycelium formation is associated with a defined sequence of events. During this process, fungi go through morphological changes associated with cell wall reorganization (Wendland 2001). For this reason, the effects of ApoB-derived peptides and CATH-2 were tested on swollen spores, initial hyphae and branched mycelium. To analyse the effects of peptides on swollen spores, spores were incubated for 6 h in minimal medium (MM). Following incubation, peptides were added at different concentrations and metabolic activity was analysed after a further 24-h incubation. It was found that ApoB-derived peptides, as well as CATH-2, were able to significantly affect the metabolic activity of swollen spores (Fig. 6). The effects of the peptides on initial hyphae were also analysed. To this purpose, *A. niger* N402 spores were incubated for 16 h, in order to allow germination and hyphal outgrowth. ApoB-derived HDPs were then added for a further 24 h and, at the end of the incubation, the WST-1 assay was performed to test the metabolic activity. Interestingly, all three ApoB-derived peptides were found to significantly affect metabolic activity of newly formed hyphae, while CATH-2 was effective only at the highest concentration tested (Fig. 6). When branched mycelium was analysed, r(P)ApoB_L_^Ala^ and r(P)ApoB_S_^Pro^ were found to be the most effective peptides, whereas r(P)ApoB_L_^Pro^ was found to be able to significantly reduce metabolic activity only at the highest concentration tested (40 μM). CATH-2 was, instead, found to be ineffective (Fig. 6).

### Analysis of fluorescently labelled r(C)ApoB_L_^Pro^ internalization into *A. niger* N402

**Fig. 7:**
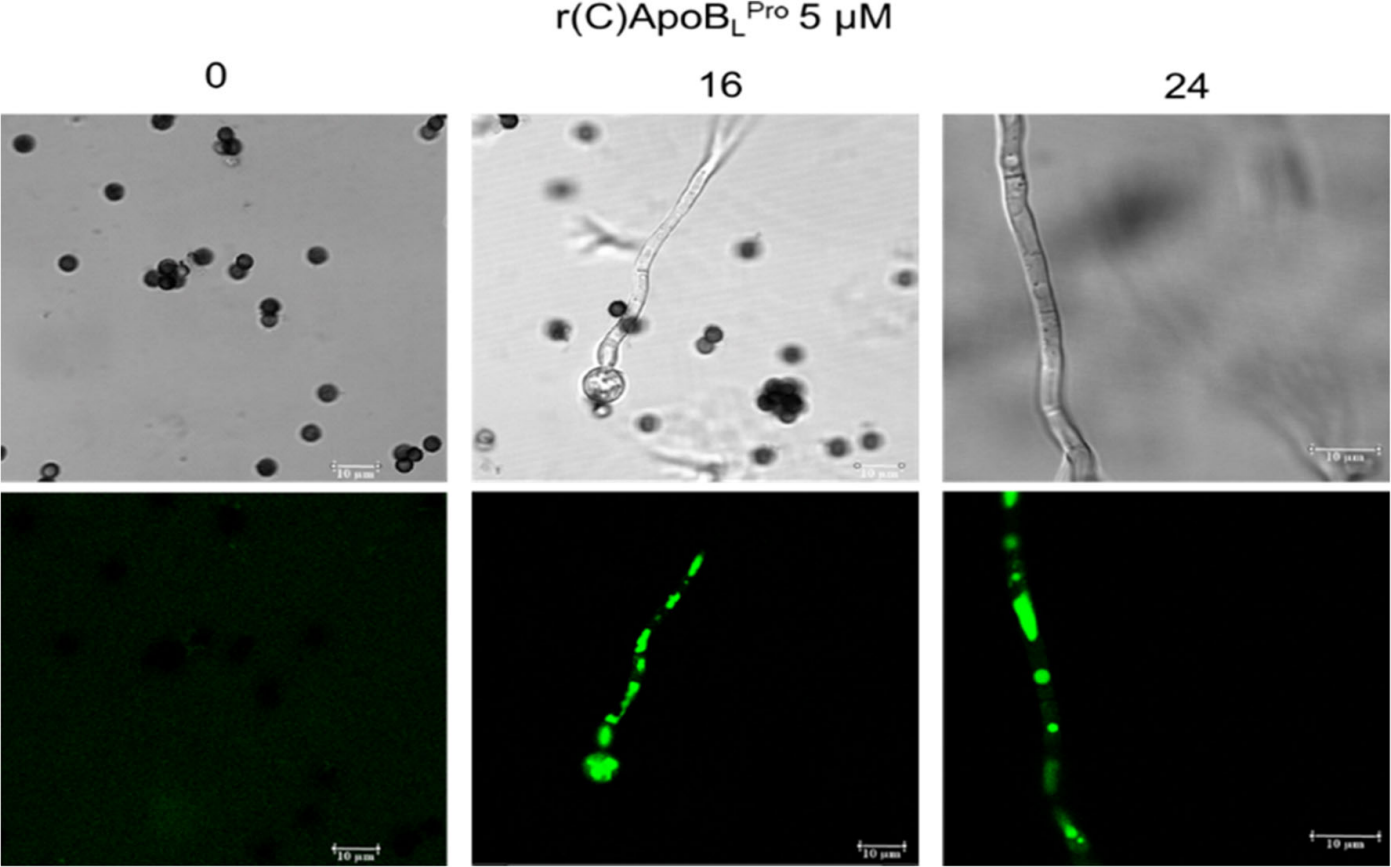
Localization of 5’-IAF-r(C)ApoB_L_^Pro^ peptide in *A. niger* N402 initial hyphae and branched mycelium. Images were acquired upon 0, 16 and 24 h incubation of *A. niger* N402 with 5’-IAF-r(C)ApoB_L_^Pro^ peptide. Scale bar 10 μm

In order to further characterize the effects of ApoB-derived peptides on *A. niger* N402, spores were incubated with 10-μM fluorescently labelled r(C)ApoB_L_^Pro^ for 24 h at 30 °C. Following incubation, confocal laser scanning microscopy analyses highlighted the ability of the 5’-IAF-r(C)ApoB_L_^Pro^ peptide to interact with swollen spores. Indeed, upon 16-h incubation, 5’-IAF-r(C)ApoB_L_^Pro^ was found to accumulate into initial hyphae, with a consequent accumulation into branched mycelium upon 24-h incubation (Fig. 7).

## Discussion

Yeasts and fungi are widespread organisms able to grow even in harsh conditions (Gulis and Bärlocher 2017; Bahafid et al. 2017). It is increasingly acknowledged that the intensive and increasing employment of antifungals in modern medicine, agriculture, and animal production is responsible for the fast development of resistance phenotypes (Kontoyiannis 2017). Along with antifungals misuse, the restricted number of effective antifungal agents represents an urgent issue (Verweij et al. 2016). The development of novel effective antifungal strategies based on yet unexplored molecules with alternative mechanisms of action is imperative. In this scenario, HDPs have attracted great attention because of their broad spectrum of activities and peculiar mechanism of action (Hancock and Lehrer 1998; Zasloff 2002; Brogden et al. 2003).

### ApoB-derived peptides exert fungicidal activity on *C. albicans* ATCC 10231

Here, the antifungal activity of ApoB-derived peptides has been evaluated for the first time on *C. albicans* ATCC 10231 and on *A. niger* N402 filamentous fungus, selected as prototypes of fungal species responsible for human infections and food spoilage, respectively. Indeed, while *Candida* species are the most common pathogens responsible for infections in hospitalized patients characterized by a high mortality rate (Strollo et al. 2017), *A. niger* is a saprophytic and filamentous fungus generally able to adapt to several habitats and to produce mycotoxins, thus representing the main contaminant of several food samples, such as fruits, vegetables, cereals and nuts leading to their discoloration, rotting and decay (Morita and Nozawa 1985; Kim and Park 2012; Roemer and Krysan 2014; Cowen et al. 2015; Prakash et al. 2015; Kumar et al. 2017). Interestingly, r(P)ApoB_L_^Pro^, r(P)ApoB_L_^Ala^ and r(P)ApoB_S_^Pro^ peptides were found to be able to exert significant fungicidal activity when tested on *C. albicans* ATCC 10231. In particular, peptides were found to exploit their fungicidal action towards *C. albicans* within 10 min when tested at their MFC concentration values. Accordingly, membrane permeabilization assays highlighted an almost immediate interaction between ApoB-derived peptides and *C. albicans* cells, resulting in damage and permeabilization of membranes. In all cases, similar effects were observed for all three ApoB-derived peptides.

### ApoB-derived peptides immediately interact with *C. albicans* ATCC 10231 cell surface

The production of fluorescently labelled r(C)ApoB_L_^Pro^ peptide allowed us to analyse peptide mechanism of action by live imaging confocal laser scanning microscopy. Fluorescently labelled r(C)ApoB_L_^Pro^ immediately localized on fungal cell surfaces, with an almost concomitant uptake of propidium iodide into the fungal cells. This phenomenon was found to progressively increase over time. Similar observations have been reported for different HDPs, such as astacidin 1 identified in hemocyanin of the freshwater crayfish *Pacifastacus leniusculus* (Choi and Lee 2014). This peptide was found to exert significant antifungal activity using a pore-forming mechanism on *C. albicans* cell membranes (Choi and Lee 2014). A similar mechanism of action has also been reported for HDPs CATH-2 and LL-37 (Ordonez et al. 2014). All these peptides have been reported to induce ATP leakage after 5-min incubation when tested on *C. albicans* cells. In earlier studies, fluorescence microscopy analyses revealed that CATH-2 is able to immediately interact with *C. albicans* cells, while LL-37 requires about 3-min incubation prior to interaction with membranes (Ordonez et al. 2014).

### ApoB-derived peptides affect *A. niger* N402 metabolic activity

Here, we also demonstrated that ApoB-derived peptides are able to affect the metabolic activity of *A. niger* N402 filamentous fungus. Interestingly, analyses on swollen spores, initial hyphae and branched mycelium highlighted that ApoB-derived peptides are able to interact more efficiently with swollen spores and hyphae than with branched mycelium. These observations are in agreement with previous findings regarding the effects of Skh-AMP 1 peptide on *Aspergillus fumigatus* (Khani et al. 2020). Indeed, Skh-AMP 1 was found to be able to affect spores’ survival rate, although at concentrations significantly higher than those required in the case of ApoB-derived peptides to exert significant effects. It has to be noticed that, differently from ApoB-derived peptides, Skh-AMP 1 was found to act on *Aspergillus* hyphae membranes more efficiently than on spores (Khani et al. 2020). In the case of KK14 de novo synthesized peptide and its analogues, a significant inhibition of the growth of *A. niger* dormant spores was demonstrated, even if all the peptide analogues were found to lost their activity when tested on the germinating conidia (Thery et al. 2019b). However, these peptides are characterized by a different mechanism of action when compared to ApoB-derived HDPs, being able to permeabilize the cell wall of *Fusarium culmorum* (Thery et al. 2019b)*.* In the case of ApoB-derived HDPs, no significant PI uptake and ATP leakage were detected upon incubation of *A. niger* incubation with peptides (data not shown). Hence, the obtained findings suggest that, even if ApoB-derived HDPs interact with the *A. niger* cell wall, no permeabilization is induced upon interaction. Consequently, peptide uptake might occur during swelling of the spores. Indeed, during this phase, water and nutrient uptake by spores is associated with a lower rigidity of the cell wall, an event that might favour peptide uptake with a consequent inhibition of fungal metabolic activity.

### ApoB-derived peptides affect different stages of growth of *A. niger* N402

Our findings highlight the huge potentiality of ApoB-derived peptides, which are able to act at low concentrations and even on different fungal stages of growth, such as spores, generally recalcitrant to the treatment with conventional antifungal agents. Interestingly, confocal laser scanning microscopy analyses, performed using fluorescently labelled r(C)ApoB_L_^Pro^ peptide, revealed peptide interaction with swollen spores and its subsequent accumulation into hyphae. In the case of PepBiotics CR173 and CR183, the ability to inactivate hyphae of *A. fumigatus* has been reported and correlated to a putative effect on mitochondria (van Eijk et al. 2020). In the literature, plant defensins, extracted from chopea seeds, have been reported to exert strong antifungal effects on *Fusarium culmorum* (Schmidt et al. 2019), although MFC values are higher than those here described for ApoB-derived peptides. In the case of chopea-thionin II, an initial interaction of the peptide with fungal cells was reported (Schmidt et al. 2019), with consequent membrane permeabilization and cell lysis or, as demonstrated here for ApoB-derived peptides, with subsequent internalization into fungal swollen spores or initial hyphae. Peptides isolated from *Leuconostoc mesenteroides* DU15 were, instead, found to be able to affect by 50% the growth of *A. niger* by causing significant morphological changes of branched mycelium, as evidenced by scanning electron microscopy analyses, and a reduction in the number of fungus cells (Muhialdin et al. 2015). Based on the obtained results, ApoB-derived peptides are able to exert strong effects on *A. niger* N402, and, even more importantly, they possess additional properties with respect to previously identified antifungal peptides, such as lower MFC values and the ability to affect different stages of fungal growth. Further experiments will be surely performed in the future to deepen on the molecular mechanism underlying the interesting properties demonstrated in the case of ApoB-derived peptides.

Altogether, the obtained findings indicate that ApoB-derived peptides represent novel antifungal agents suitable for the future development of effective strategies to treat fungal infections generally recalcitrant to conventional therapeutic approaches, also considering that they have been previously demonstrated to be neither toxic nor haemolytic for murine and human eukaryotic cell lines (Gaglione et al. 2017).

## Supplementary Information

ESM 1 (PDF 915 kb)

ESM 2 (MOV 690 kb)

## Data Availability

All data generated or analysed during this study are included in this published article and its supplementary information files.
