# Supplementary material for: Host defence peptides identified in human apolipoprotein B as promising antifungal agents
Source: Appl Microbiol Biotechnol. 2021 Feb 12;105(5):1953–64. doi: 10.1007/s00253-021-11114-3 (PMC7907042; doi:10.1007/s00253-021-11114-3)
Supplement: Supplementary file 1 — (PDF 915 kb) [file 253_2021_11114_MOESM1_ESM.pdf]

Journal Name: Applied Microbiology and Biotechnology

Manuscript title: Host Defence Peptides identified in human Apolipoprotein B as promising antifungal agents

Eliana Dell'Olmo[1,2], Rosa Gaglione[1,3], Angela Cesaro[1], Valeria Cafaro[4], Wieke R. Teertstra[5], Hans de Cock[5], Eugenio Notomista[4], Henk P. Haagsman[2], Edwin J.A. Veldhuizen[2], Angela Arciello[1,3].

[1]Department of Chemical Sciences, University of Naples Federico II, 80126 Naples, Italy;

[2]Department of Biomolecular Health Sciences, Division of Infectious Diseases and Immunology, Section Molecular Host Defence, Faculty of Veterinary Medicine, Utrecht University, Utrecht, The Netherlands;

[3]Istituto Nazionale di Biostrutture e Biosistemi (INBB), Rome, Italy;

[4]Department of Biology, University of Naples Federico II, 80126 Naples, Italy;

[5]Molecular Microbiology, Department of Biology, Faculty of Science, Utrecht University, Utrecht, The Netherlands.

Correspondence: Edwin J.A. Veldhuizen, Department of Biomolecular Health Sciences, Division of Infectious Diseases and Immunology, Section Molecular Host Defence, Faculty of Veterinary Medicine, Utrecht University, Utrecht, The Netherlands, tel.: 0302535361, e-mail: e.j.a.veldhuizen@uu.ni; Angela Arciello, Department of Chemical Sciences, University of Naples Federico II, 80126 Naples, Italy, tel.: +39 081679147, e-mail: anarciel@unina.it.

Running title: ApoB-derived peptides as antifungal agents

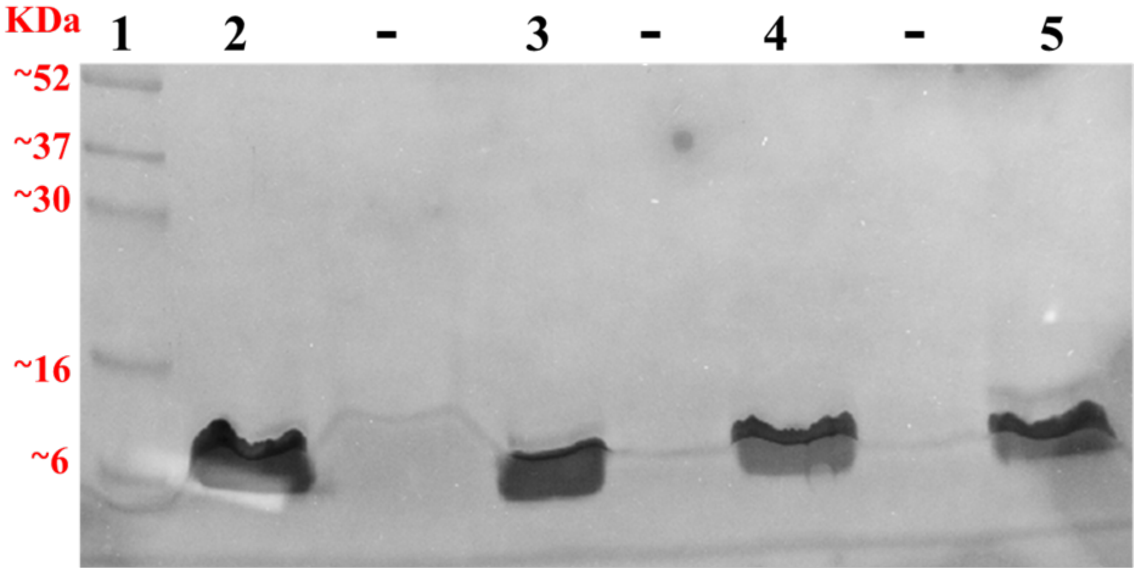

Supplementary Figure S1. Analysis of recombinant purified peptides by 18% SDS-PAGE. Lane 1: pre-stained markers; lane 2: r(P)ApoB$_L^{Pro}$ peptide (20 μg); lane 3: r(P)ApoB$_S^{Pro}$ peptide (20 μg); lane 4: r(P)ApoB$_L^{Ala}$ peptide (20 μg); lane 5: IAF-r(C)ApoB$_L^{Pro}$ peptide (20 μg).

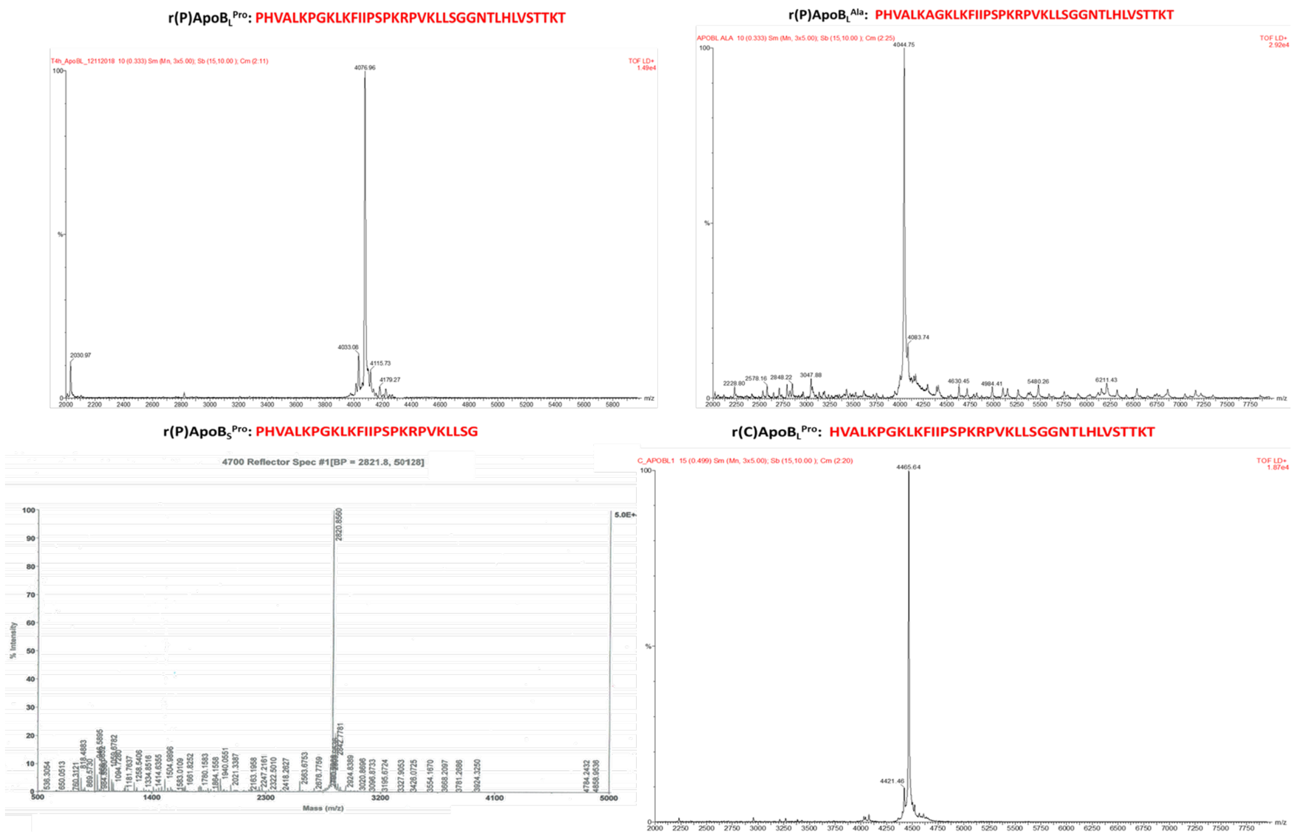

Supplementary Figure S2. Analysis of recombinant purified peptides by MALDI-TOF mass spectrometry.

Supplementary Table S1. MFC_{100} and MFC_{50} values of peptides under study towards *Candida albicans* ATCC 10231 and *Aspergillus niger* N402, respectively.

Peptide	*Candida albicans* ATCC 10231 MFC_{100} (μM)	*Aspergillus niger* N402 MFC_{50}(μM)
r(P)$ApoB_L^{Pro}$	10	10
r(P)$ApoB_L^{Ala}$	10	2.5
r(P)$ApoB_S^{Pro}$	20	2.5
IAF-r(C)$ApoB_L^{Pro}$	10	5
CATH-2	20	2.5

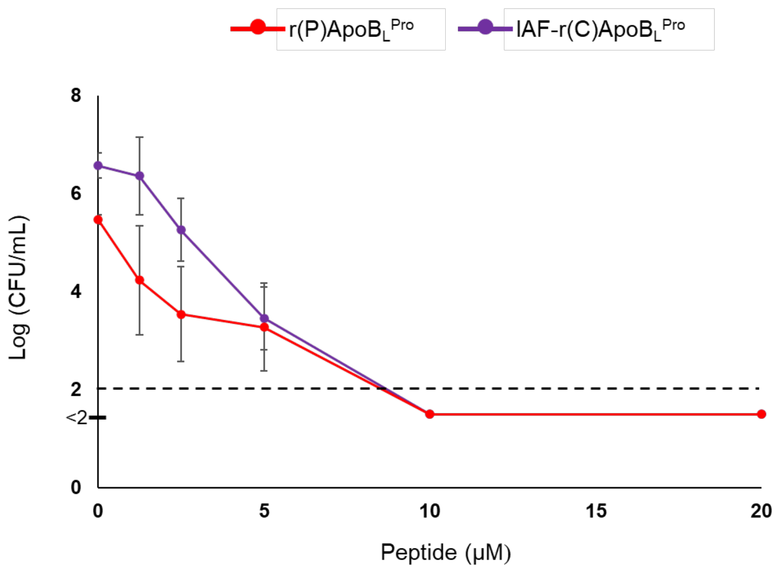

Supplementary Figure S3. Antifungal activity of 5'-IAF-r(C)ApoB$_L^{Pro}$ towards C. albicans ATCC 10231cells. Minimum fungicidal concentration (MFC) value was assessed by colony count assays. Data represent the mean (± SEM) of three independent experiments, each one carried out with triplicate determinations.

Supplementary movie. Tracking of 5'-IAF-r(C)ApoB$_L^{Pro}$ internalization in *C. albicans* ATCC 10231 by live cell imaging.
